# Case Report: Guillain−Barré syndrome temporally associated with levofloxacin exposure and improvement following efgartigimod treatment

**DOI:** 10.3389/fimmu.2025.1729694

**Published:** 2026-01-07

**Authors:** Shiyuan Xie, Yueling Zhang, Feng Shen, Lining Wu, Yunxia Tang, Jun Li

**Affiliations:** 1 Department of Pharmacy, The Second Affiliated Hospital of Guangxi Medical University, Nanning, China; 2 Department of Neurology, The Second Affiliated Hospital of Guangxi Medical University, Nanning, China

**Keywords:** efgartigimod, FcRn inhibitor, immunoglobulin, levofloxacin, methylprednisolone, plasma exchange

## Abstract

Levofloxacin, the L-isomer of the racemic fluoroquinolone ofloxacin, is a broad-spectrum antibiotic with broad clinical applications in both prophylactic and therapeutic indications. However, it has been associated with various adverse reactions, including Guillain−Barré syndrome (GBS). GBS is an immune-mediated peripheral neuropathy that typically presents with acute-onset symmetrical flaccid paralysis. Here, we report a case of GBS temporally associated with levofloxacin exposure that improved after initiation of efgartigimod, following limited response to plasma exchange, methylprednisolone, and intravenous immunoglobulin. To our knowledge, there are no previously published cases of GBS associated with levofloxacin exposure treated with efgartigimod therapy. This case suggests that efgartigimod may represent a promising treatment option for GBS temporally associated with levofloxacin exposure. However, this single case cannot establish efficacy, and delayed responses to prior therapies or spontaneous recovery remain possible alternative explanations. Further studies with larger cohorts are needed to clarify the mechanisms underlying this therapy.

## Introduction

Guillain−Barré syndrome (GBS) is an immune-mediated peripheral neuropathy, with acute-onset symmetrical flaccid paralysis as its main clinical manifestation (1). GBS encompasses several clinical subtypes, including Miller fisher syndrome, acute inflammatory demyelinating polyradiculoneuropathy, and acute motor axonal neuropathy (AMAN) (2). More than 20% of patients with GBS experience poor outcomes or death, even when managed with timely treatment (3). Levofloxacin, the L-isomer of the racemic fluoroquinolone ofloxacin, is a broad-spectrum antibiotic with diverse clinical applications in both prophylactic and therapeutic indications (4, 5). Several reports have suggested a possible association between levofloxacin therapy and subsequent onset of GBS (5). Efgartigimod has demonstrated safety and efficacy in treating generalized myasthenia gravis patients with acetylcholine receptor (AChR) antibody positivity (Ab+) (6). In addition, the therapeutic potential of efgartigimod in GBS is supported by several studies (7–10).

Herein, we report a case of GBS occurring in a 23-year-old female patient with urinary tract infection who developed muscle weakness of the extremities after treatment with levofloxacin. Using the Naranjo probability scale, we identified levofloxacin as the probable cause of muscle weakness (11). On this basis, and supported by electromyography and serum analyses, she was diagnosed with GBS temporally associated with levofloxacin exposure. Clinical improvement was observed after initiation of efgartigimod, following limited response to plasma exchange (PLEX), methylprednisolone, and intravenous immunoglobulin (IVIG) therapies. A literature search was conducted through PubMed, Cochrane Library, ClinicalTrials, and EMBASE databases, covering publications up to November 20, 2025. The following combined text words and MeSH terms were used: “Guillain−Barré syndrome”, “levofloxacin”, “ofloxacin”, “fluoroquinolones”, and “efgartigimod”. To our knowledge, efgartigimod has not previously been reported in the treatment of GBS temporally associated with levofloxacin exposure. However, other similar cases may exist but be unpublished or non-indexed.

## Case report

**Figure 1:**
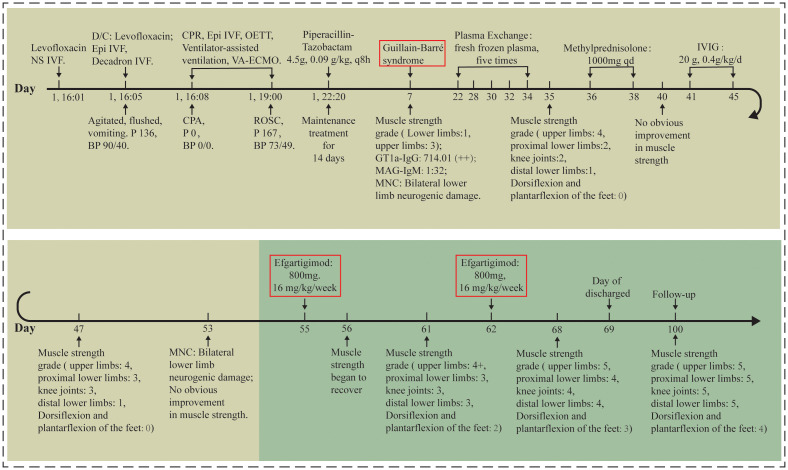
The treatment procedure of the patient. Day 1: the day of admission to our emergency department. NS IVF, normal saline intravenous fluid; D/C: discontinue; Epi, Epinephrine; P, pulse; BP, blood pressure; CPR, cardiopulmonary resuscitation; OETT, oral endotracheal intubation; VA-ECMO, venous-arterial extracorporeal membrane oxygenation; CPA, cardiopulmonary arrest; ROSC, return of spontaneous circulation; MNC, motor nerve conduction velocity; IVIG, intravenous immunoglobulin.

A 23-year-old female presented to a clinic with symptoms of urinary frequency, urgency, and dysuria. She was diagnosed with a urinary tract infection and received intravenous levofloxacin sodium chloride injection (0.5 g levofloxacin + 0.9 g sodium chloride in 100 mL solution) at an infusion rate of 45 drops/min. After approximately one-third of the infusion had been administered, she developed palpitations, dyspnea, facial flushing, restlessness, and vomiting, with a heart rate of 136 bpm and blood pressure of 90/40 mmHg. The infusion was immediately discontinued, and she received 1 mg epinephrine and 10 mg dexamethasone in 100 mL saline intravenously. However, 5 minutes later, she experienced cardiopulmonary arrest. She was transferred to our emergency department (Day 1; **Figure 1**). Emergency resuscitation, including cardiopulmonary resuscitation, epinephrine, propofol, endotracheal intubation, and VA-ECMO, was initiated. She regained heart rate and blood pressure (73/49 mmHg, HR:167 bpm). The bacterial culture of the patient’s urine showed the presence of Escherichia coli. The urinalysis results included white blood cells (+3), microscopic pus cells (2+), and nitrite (+). Therefore, piperacillin/tazobactam (4.5 g, 0.09 g/kg every 8 hours) was administered intravenously for 14 days as antimicrobial therapy.

On Day 7, the patient exhibited diminished tendon reflexes and a sensory level at the costal margin. Electromyogram evidence revealed bilateral lower limb neuropathic damage (**Supplementary Figure 2-1**). Laboratory findings showed elevated blood ganglioside antibody levels: anti-GT1a-IgG (serum) 714.01 (++), and anti-myelin-associated glycoprotein antibody (MAG-IgM) (serum): 1:32. Neurological examination further demonstrated disorientation to person. Motor strength was impaired, with upper limb muscle strength of grade 3/5 and lower limb strength of grade 1/5. According to the Naranjo Adverse Drug Reaction (ADR) probability scale, levofloxacin was the probable cause of the muscle weakness (total score: 13; causality classification: ≥ 9, Definite; 5–8, Probable; 1–4, Possible; ≤ 0, Doubtful). Nerve conduction studies showed decreased compound muscle action potential (CMAP) amplitude and abnormal F-waves in both common peroneal and tibial nerves. Terminal latency, motor conduction velocity, sensory conduction velocity, and sensory nerve action potential were normal. These findings are consistent with axonal motor abnormalities (Day 7; **Table 1**). As shown in **Table 2**, the patient’s condition was severe, with a GBS Disability Scale score of 4/6 and a Manual Muscle Testing (MMT) summary score of 1/5 (Day 7). Based on the MMT results, the Naranjo ADR probability scale score, antibody data, and electrodiagnostic evidence, the patient was diagnosed with GBS temporally associated with levofloxacin exposure. Furthermore, the GBS subtype in this case was most likely AMAN.

**Table 1:** Nerve conduction study findings in the patients.

Nerve		Day7					Day53				
		CMAP (mV)/ SNAP (uV) amplitude	DML (ms)	MCV/SCV (m/s)	F wave latency (ms)	Frequency of F wave	CMAP (mV)/ SNAP (uV) amplitude	DML (ms)	MCV/SCV(m/s)	F wave latency (ms)	Frequency of F wave
Median	Left	NT	NT	NT	NT	NT	15.8/28.9	2.5	54/56	23	70%↓
	Right	10.1/19.7	2.5	54/67	23.4	12.5%↓	12.9/31.6	2.9	56/58	21.3	100%
Ulnar	Left	NT	NT	NT	NT	NT	11.4/13.7	2	55/57	22.1	100%
	Right	9.3/20.2	1.9	52/58	22.1	68.8%↓	12.7/12	2.2	54/55	20.4	100%
Tibial	Left	0.3↓/16.4	4	46/61	NR	NR	NR/17.7	NR	46/69	NR	NR
	Right	1.0↓/17.2	4.3	48/48	NR	NR	NR/8.4↓	NR	NR/58	NR	NR
Common Peroneal	Left	1.0↓/6.4	4.3	54/55	NT	NT	0.5↓/4.3	3.6	58/65	NT	NT
	Right	NR/1.6	NR	NR/79	NT	NT	NR/NR	NR	NR/NR	NT	NT

CMAP compound motor action potential; SNAP sensory nerve action potential; DML distal motor latency; MCV motor conduction velocity; SCV sensory conduction velocity; ↓ decrease; ↑ increase; NT not tested; NR non-responsive.

**Table 2:** The changes of the GBS disability score and manual muscle testing*.

Timeline	Treatment or follow-up	GBS disability scale	Manual muscle testing
Day 7	Tracheal extubation (day 6)	4	1
Day 35	Plasma exchange (day 22, 28, 30, 32, 34)	4	1
Day 40	Methylprednisolone (day36 -38)	4	1
Day 47	Immunoglobulin (day 41-45)	4	2
Day 61	Efgartigimod (day 55)	4	3
Day 68	Efgartigimod (day 62)	3	4
Day 100	Follow-up	1	4

*GBS Disability Scale (0–6 Score): 0, a healthy state; 1, minor symptoms and capable of running; 2, able to walk 10 m or more without assistance but unable to run; 3, able to walk 10 m across an open space with help; 4, bedridden or chairbound; 5, requiring assisted ventilation for at least part of the day; 6, dead. Manual Muscle Testing (0–5 grade): 0, no muscle contraction (complete paralysis); 1, flicker or trace of contraction; 2, full range of motion (ROM) with gravity eliminated; 3, full ROM against gravity, no resistance; 4, full ROM against gravity and moderate resistance; 5, full ROM against gravity and maximum resistance (normal strength).

Initially, five cycles of PLEX were administered. The replacement fluid was fresh frozen plasma. The plasma volumes exchanged were as follow: 1,980 mL (Day 22), 1,980 mL (Day 28), 2,010 mL (Day 30), 2,000 mL (Day 32), and 1,990 mL (Day 34). Following PLEX therapy, physical examination revealed improved orientation to person and logical thought processes compared to baseline. Color discrimination was preserved, though the patient still demonstrated a persistent lack of insight. Motor function demonstrated a pattern of severe residual weakness, with upper limbs at grade 4/5, proximal lower limbs at grade 2/5, knee joints at grade 2/5, distal lower limbs at grade 1/5, and dorsiflexion and plantarflexion of the feet at grade 0/5. Given the poor response to PLEX, methylprednisolone pulse therapy (MPPT; 1,000 mg once daily) was administered for 3 days. Due to the lack of improvement with MPPT, IVIG therapy was initiated. The IVIG regimen was 0.4 g/kg/day for 5 days (total dose: 20 g daily). Post-treatment assessment demonstrated partial improvement in motor function, but persistent distal weakness remained: upper limbs were grade 4/5, proximal lower limbs grade 3/5, knee joints grade 3/5, distal lower limbs grade 1/5, and dorsiflexion and plantarflexion of the feet grade 0/5. By Day 53, nerve conduction studies revealed partial recovery, with the F-wave frequency in the bilateral median and ulnar nerves returning toward normal. In contrast, the CMAP amplitude in the bilateral common peroneal and tibial nerves further decreased compared to Day 7 (**Table 1**).

Subsequently, efgartigimod was administered for 2 weeks at a weekly dose of 800 mg (16 mg/kg). As shown in **Table 2**, after completion of the first week of treatment (Day 61), the patient’s GBS Disability Scale score remained 4/6 and the MMT total score was assessed at 3/5. Moreover, motor function demonstrated further recovery, with upper limbs at grade 4+/5, proximal lower limbs at grade 3/5, knee joints at grade 3/5, distal lower limbs at grade 3/5, and dorsiflexion and plantarflexion of the feet at grade 2/5. Functional milestones included assisted standing and slow ambulation with support. Upon completing the second week of efgartigimod (Day 68), the patient’s GBS Disability Scale score was 3/6 and the overall MMT score was 4/5. Further enhanced mobility was observed: independent sit-to-stand, slow assisted walking, upper limbs at grade 5/5, proximal lower limbs at grade 4/5, knee joints at grade 4/5, distal lower limbs at grade 4/5, and dorsiflexion and plantarflexion of the feet at grade 3/5. Neurological evaluation showed persistent T10 sensory deficits, normal upper limb reflexes (++), absent lower limb reflexes (–), Brunnstrom stage at grade 3/6 for lower limbs, Holden walking ability at grade 2/5, and Activities of Daily Living score at 55/100. At discharge, the patient achieved independent ambulation but exhibited residual memory and calculation deficits with preserved orientation. At Day 100 of follow-up, the patient showed sustained ambulation capability and near-complete motor recovery, with upper limbs at grade 5/5, proximal lower limbs at grade 5/5, knee joints at grade 5/5, distal lower limbs at grade 5/5, and dorsiflexion and plantarflexion of the feet at grade 4/5. Moreover, the GBS Disability Scale score was 1/6 and the MMT total score was 4/5.

## Discussion

Fluoroquinolones are a class of broad-spectrum antibacterial drugs with extensive prophylactic and therapeutic indications covering gastrointestinal, genitourinary, respiratory, ophthalmic and bone infections (5). Levofloxacin, the L-isomer of the racemic fluoroquinolone ofloxacin, exerts its antimicrobial effects by inhibiting DNA gyrase and topoisomerase IV (4). The most frequently reported side effects of levofloxacin are gastrointestinal adverse effects (4). Beyond gastrointestinal symptoms, neurological disturbances are the next most common side effects (12, 13). Sensorimotor axonal neuropathy has been documented during fluoroquinolones treatment (14). Furthermore, peripheral neuropathy has been formally included in product labeling and medication guides as a recognized risk associated with systemic administration of fluoroquinolones (5). GBS is an immune-mediated polyradiculoneuropathy, with neurological symptoms typically emerging in most patients following an infectious illness (15). It has been reported that GBS can be triggered by surgery, particular vaccinations and levofloxacin therapy (5, 16, 17). **Table 3** shows the characteristics of GBS reports for individual fluoroquinolones. GBS was reported for ciprofloxacin (n = 24), levofloxacin (n = 13), moxifloxacin (n = 11), and ofloxacin (n = 1). The majority of the GBS reports were directly submitted by health care professionals, with fluoroquinolones being the primary suspect in GBS occurrence. Furthermore, the majority of the GBS events indicated concurrent exposure to two or more drugs in addition to fluoroquinolones (**Table 3**). Notably, a study reported the case of a 61-year-old male patient who developed GBS after three days of ofloxacin therapy for a urinary tract infection (18) (**Table 3**). Moreover, the reports of adverse events submitted to the FDA Adverse Event Reporting System (FAERS) from 1997 to 2012 revealed that among 46,257 fluoroquinolone-related adverse events, 539 involved peripheral neuropathy (5). Within peripheral neuropathy cases, 9% were attributed to GBS, positioning GBS as the sixth most commonly reported neurological adverse event linked to fluoroquinolones (5). Autoimmune adverse effects of fluoroquinolones may be related not only to the core ring structure, but also to the side chain modifications (19). Moreover, ciprofloxacin may promote T-cell-mediated hypersensitivity via haptenization, exacerbating underlying immune dysregulation (20). Diagnosis of GBS relies on clinical presentation, supported by laboratory findings and electro­diagnostic (21). In our case, laboratory findings showed elevated serum GT1a-IgG. In conjunction with MMT, the Naranjo probability scale, and electrodiagnostic evidence, the patient was diagnosed with GBS temporally associated with levofloxacin exposure. However, the exact mechanism remains unclear, and further investigation is warranted to elucidate the specific pathways linking levofloxacin exposure to GBS.

**Table 3:** Characteristics of fluoroquinolone-related Guillain-Barré syndrome cases.

First Author	Ali AK et al. (5)*			Schmidt S et al. (18)	Our case
Drug of fluoroquinolone and number of Patient	Ciprofloxacin n = 24	Levofloxacin n = 13	Moxifloxacin n = 11	ofloxacin n = 1	Levofloxacin n = 1
Patient’s age (y)	62 (26, 90) n = 22	49 (12, 83) n = 11	52 (28, 68) n= 11	61	23
Patient’s sex					
Female	5 (20.8)	5 (38.5)	2 (18.2)	0	1
Male	17 (70.8)	7 (53.8)	9 (81.8)	1	0
Unknown	2 (8.3)	1 (7.7)	0	0	0
Event outcome					
Death	2 (8.3)	0	1 (9)	0	0
Disability	3 (12.5)	2 (15.4)	0	1	1
Reporting source					
Health care professional	13 (54.2)	8 (61.5)	5 (45.4)	1	1
Consumer	0	1 (7.7)	0	0	0
Manufacturer	0	0	2 (18.2)	0	0
Clinical study	0	0	1 (9)	0	0
Other/unknown	11 (45.8)	4 (30.8)	3 (27.3)	0	0
Drug role in event occurrence					
Primary suspect	22 (91.7)	12 (92.3)	10 (91)	1	1
Secondary suspect	2 (8.3)	1 (7.7)	0	0	0
Concomitant	0	0	1 (1)	0	0
Onset of event (d)y	3 (0, 26) n =7	4 (0, 7) n = 9	2 (0, 9) n = 6	3	1
Number of concomitant drugs					
0	11 (45.8)	3 (23.1)	3 (27.3)	1	0
1	5 (20.8)	3 (23.1)	0	0	0
≥2	8 (33.3)	7 (53.8)	8 (72.7)	0	1

*Values reported as Median (min, max) and number or frequencies (percentages) of reports with valid information.

Current immunotherapeutic options for GBS encompass PLEX, IVIG, and glucocorticoids (8). PLEX may demonstrate clinical efficacy when initiated within the first week of treatment; however, it carries risks such as immune complex depletion, cytokine removal, and complications including arrhythmias or hypotension (22, 23). IVIG, a biological agent derived from pooled plasma donations (2,000−16,000 healthy donors), is associated with transient side effects in most patients (e.g., tachycardia, headaches, vomiting, nausea). Severe adverse events occur in 2−6% of cases and may include renal impairment, thrombosis, neutropenia, or aseptic meningitis (24, 25). Although intravenous glucocorticoids are occasionally considered for complex or critically ill patients, their use remains limited to case-by-case evaluations (22). Despite these interventions, many GBS patients experience prolonged residual symptoms, significantly impairing quality of life (3). This underscores the urgent need for the development of targeted immunotherapies with novel mechanisms of action to improve treatment strategies.

Efgartigimod, a rationally engineered molecule with high binding affinity for the neonatal Fc receptor (FcRn), represents an effective and well-tolerated therapeutic option for the management of autoimmune diseases (7, 26). Efgartigimod is a recombinant human IgG1 Fc fragment engineered while preserving its pH-dependent binding characteristics (8, 9). By competitively displacing endogenous IgG from FcRn, this agent disrupts the IgG recycling pathway, thereby accelerating IgG degradation and reducing pathogenic autoantibody levels (26). On December 17, 2021, the FDA granted approval for efgartigimod to treat adult patients with anti-acetylcholine receptor (AChR) antibody-positive generalized myasthenia gravis. Moreover, on June 24, 2024, the FDA approved efgartigimod for the treatment of chronic inflammatory demyelinating polyneuropathy in adults. For this indication, the therapy is administered subcutaneously as weekly injections. Numerous clinical trials and case studies have investigated efgartigimod in various inflammatory conditions driven by pathogenic autoantibodies, including GBS and inflammatory myositis (8, 27, 28). Furthermore, evidence indicates that FcRn blockade may represent a unique therapeutic approach for GBS. Prior studies have suggested that efgartigimod monotherapy shows promise in this setting, highlighting its potential as a new treatment option (7, 9, 10, 27, 28). These findings support the potential of efgartigimod to prevent further nerve injury and slow disease progression in GBS. Additionally, a phase II randomized, double-blind, controlled clinical trial (NCT05701189, registered on ClinicalTrials.gov) is currently underway to compare the safety and effectiveness of efgartigimod and IVIG as standalone treatments for GBS; its results may offer further insights. Therefore, FcRn antagonists hold considerable promise as alternatives to conventional therapies such as PLEX, IVIG, or glucocorticoids.

In the present case, PLEX therapy yielded only limited improvement in limb muscle strength. Furthermore, MPPT produced no significant benefit, suggesting the ineffectiveness of glucocorticoid treatment. Subsequent IVIG therapy also resulted in minimal improvement. Notably, clinical recovery was observed after initiation of efgartigimod, underscoring its potential as an emerging therapeutic alternative for GBS temporally associated with levofloxacin exposure. However, a single case cannot establish efficacy, and delayed responses to prior therapies or spontaneous recovery may represent alternative explanations. In addition, the follow-up in this case is limited. Given the currently scarce evidence on efgartigimod in GBS temporally associated with levofloxacin exposure, further studies are required to elucidate the specific mechanisms underlying this therapy.

## Conclusion

We report a case of GBS temporally associated with levofloxacin exposure, in which clinical improvement was observed after initiation of efgartigimod, following limited responses to PLEX, methylprednisolone, and IVIG. The findings suggest that efgartigimod may be a promising new treatment option for GBS temporally associated with levofloxacin exposure, warranting further investigation in studies with larger sample sizes.

## Data Availability

The original contributions presented in the study are included in the article/**Supplementary Material**. Further inquiries can be directed to the corresponding authors.
