# Supplementary material for: Case Report: Guillain−Barré syndrome temporally associated with levofloxacin exposure and improvement following efgartigimod treatment
Source: Front Immunol. 2026 Jan 7;16:1729694. doi: 10.3389/fimmu.2025.1729694 (PMC12819696; doi:10.3389/fimmu.2025.1729694)
Supplement: Supplementary file 1 [file DataSheet1.pdf]

ID #: 242200

The Second Affiliated Hospital of Guangxi Medical University
the Report on Electromyographic Evoked Potential（Day7）

Name：　Sex：Female　Age：23 y

ID：242200　Department：EICU

Right　Tibial　MNC　# 8
Rec: AH, Stim: Ankle, Popliteal fossa　Record　16:58:23

Switch: STOP Stimulator: 1 | Rate: 1 Hz | Level: 27.4 mA | 0.2 ms | Single
Step: Hold | Average: Off | Sig. Enhancer: Off

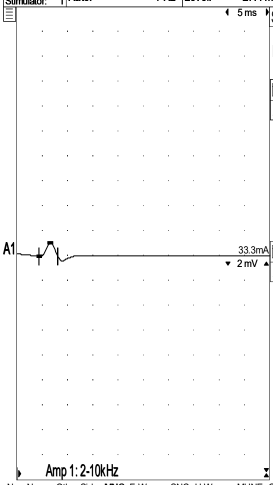

Recording Site: Abductor hallucis

Stimulus Site	Lat1 ms	Dur ms	Amp mV	Area mVms
A1: Ankle	4.3	3.7	1.0	1.7

Segment	Dist mm	Diff ms	CV m/s
Abductor hallucis-Ankle		4.3	

New Nerve　Other Side　MNC　F-Waves　SNC　H-Waves　MUNE　CVD　20.1

Right　Peroneal　MNC　#10
Rec: EDB, Stim: Ankle, Fibular head, PF　Record　16:58:28

Switch: STOP Stimulator: 1 | Rate: 1 Hz | Level: 27.4 mA | 0.2 ms | Single
Step: Hold | Average: Off | Sig. Enhancer: Off

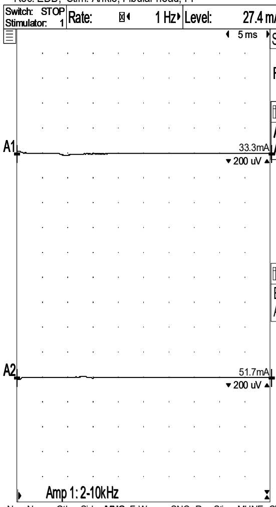

Recording Site: Extensor digitorum brevis

Stimulus Site	Lat1 ms	Dur ms	Amp mV	Area mVms
A1: Ankle				
A2: Fibula (head)				

Segment	Dist mm	Diff ms	CV m/s
Extensor digitorum brevis-Ankle			
Ankle-Fibula (head)			

New Nerve　Other Side　MNC　F-Waves　SNC　Rep Stim　MUNE　CVD　20.1

Left　Tibial　MNC　#15
Rec: AH, Stim: Ankle, Popliteal fossa　Record　16:58:34

Switch: STOP Stimulator: 1 | Rate: 1 Hz | Level: 27.4 mA | 0.2 ms | Single
Step: Hold | Average: Off | Sig. Enhancer: Off

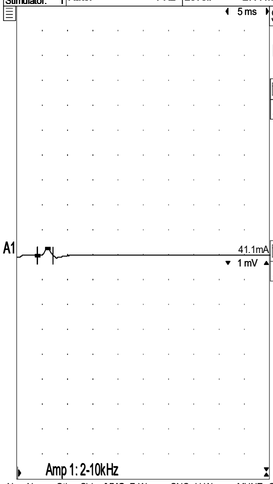

Recording Site: Abductor hallucis

Stimulus Site	Lat1 ms	Dur ms	Amp mV	Area mVms
A1: Ankle	4.0	3.1	0.3	0.4

Segment	Dist mm	Diff ms	CV m/s
Abductor hallucis-Ankle		4.0	

New Nerve　Other Side　MNC　F-Waves　SNC　H-Waves　MUNE　CVD　20.1

Left　Peroneal　MNC　#17
Rec: EDB, Stim: Ankle, Fibular head, PF　Record　16:58:38

Switch: STOP Stimulator: 1 | Rate: 1 Hz | Level: 27.4 mA | 0.2 ms | Single
Step: 2 | Average: Off | Sig. Enhancer: Off

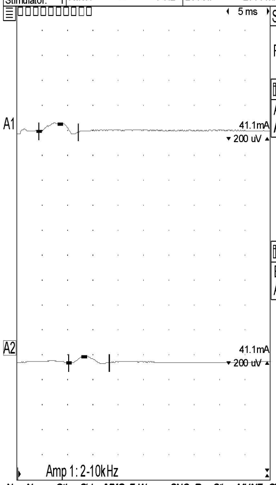

Recording Site: Extensor digitorum brevis

Stimulus Site	Lat1 ms	Dur ms	Amp mV	Area mVms
A1: Ankle	4.3	7.8	0.1	0.3
A2: Fibula (head)	10.2	8.0	0.1	0.2

Segment	Dist mm	Diff ms	CV m/s
Extensor digitorum brevis-Ankle		4.3	
Ankle-Fibula (head)	320	5.9	54

New Nerve　Other Side　MNC　F-Waves　SNC　Rep Stim　MUNE　CVD　20.1

Left Femoral MNC #22
Rec: Vastus medialis, Stim: Inguinal ligament Record 16:58:43

Switch: STOP Stimulator: 1	Rate: 1 Hz	Level: 27.4 mA	0.2 ms	Single

Step: Hold | Average: Off | Sig. Enhancer: Off

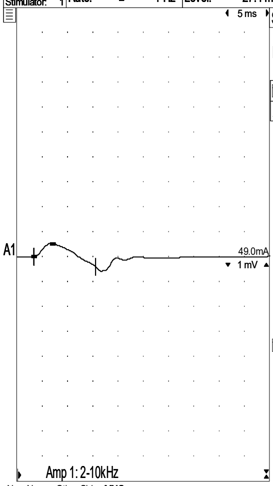

Recording Site: Vastus medialis

Stimulus Site	Lat1 ms	Dur ms	Amp mV	Area mVms
A1: Above inguinal ligam	U 8.4 3.3	12.2	0.5	3.5

New Nerve Other Side **MNC** 20.1

Right Superficial peroneal SNC #12
Rec: Dorsum of Foot, Stim: Ankle Record 16:58:53

Switch: STOP Stimulator: 1	Rate: 1 Hz	Level: 27.4 mA	0.1 ms	Single

Step: 1 | Average: On | Sig. Enhancer: Off

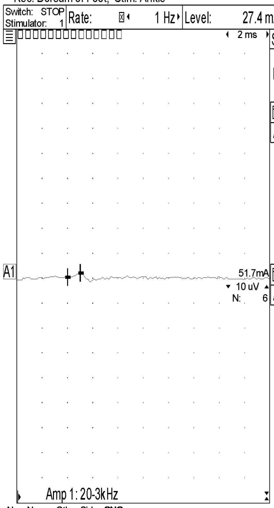

Recording Site: Fibula (head)

Stimulus Site	Lat1 ms	Lat2 ms	Amp uV
**A1: Ankle**	4.0	5.0	L10.0 1.6

Segment	Dist mm	Diff ms	CV m/s
Ankle-Fibula (head)	220	4.0	L40.0 55

New Nerve Other Side **SNC** 20.1

Right Sural SNC #13
Rec: Lat. Mal., Stim: Calf (Antidromic) Record 16:58:58

Switch: STOP Stimulator: 1	Rate: 1 Hz	Level: 27.4 mA	0.1 ms	Single

Step: 1 | Average: On | Sig. Enhancer: On

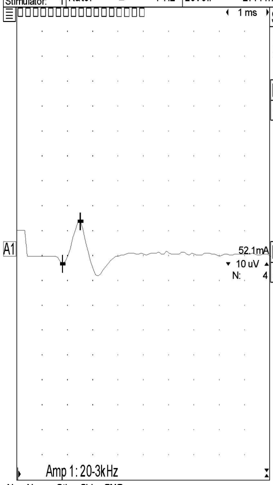

Recording Site: Soleus

Stimulus Site	Lat1 ms	Lat2 ms	Amp uV
**A1: Lateral malleolus**	1.8	2.5	L10.0 17.2

Segment	Dist mm	Diff ms	CV m/s
Soleus-Lateral malleolus	110	1.8	L41.0 61

New Nerve Other Side **SNC** 20.1

Right Saphenous SNC #14
Rec: Border of Tibia, Stim: Medial aspect knee Record 16:59:06

Switch: STOP Stimulator: 1	Rate: 1 Hz	Level: 27.4 mA	0.1 ms	Single

Step: 1 | Average: On | Sig. Enhancer: On

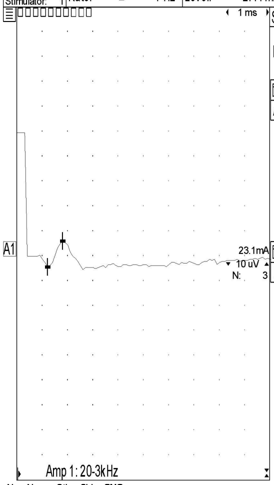

Recording Site: Lower leg

Stimulus Site	Lat1 ms	Lat2 ms	Amp uV
**A1: Knee**	U 2.8 1.2	1.8	L7.00 10.7

Segment	Dist mm	CV m/s
Lower leg-Knee	80	L54.0 67

New Nerve Other Side **SNC** 20.1

Left | Superficial peroneal | SNC | #19

Rec: Dorsum of Foot, Stim: Ankle | Record | 16:59:11

Switch: STOP | Stimulator: 1 | Rate: 1 Hz | Level: 27.4 mA | 0.1 ms | Single

Step: 1 | Average: On | Sig. Enhancer: On

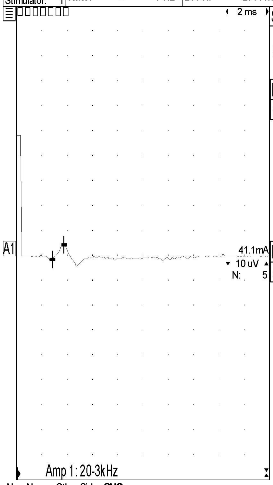

Recording Site: Fibula (head)

Stimulus Site	Lat1 ms	Lat2 ms	Amp uV
A1: Ankle	2.8	3.7	6.1 L10.0

Segment	Dist mm	Diff ms	CV m/s
Ankle-Fibula (head)	220	2.8	79 L40.0

New Nerve Other Side SNC

20.1

Left | Sural | SNC | #20

Rec: Lat. Mal., Stim: Calf (Antidromic) | Record | 16:59:15

Switch: STOP | Stimulator: 1 | Rate: 1 Hz | Level: 27.4 mA | 0.1 ms | Single

Step: 1 | Average: On | Sig. Enhancer: Off

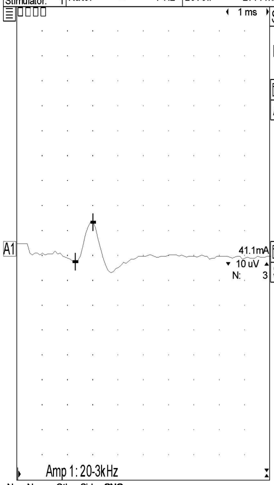

Recording Site: Soleus

Stimulus Site	Lat1 ms	Lat2 ms	Amp uV
A1: Lateral malleolus	2.3	3.0	16.4 L10.0

Segment	Dist mm	Diff ms	CV m/s
Soleus-Lateral malleolus	110	2.3	48 L41.0

New Nerve Other Side SNC

20.1

Left | Saphenous | SNC | #21

Rec: Border of Tibia, Stim: Medial aspect knee | Record | 16:59:21

Switch: STOP | Stimulator: 1 | Rate: 1 Hz | Level: 27.4 mA | 0.1 ms | Single

Step: 1 | Average: On | Sig. Enhancer: On

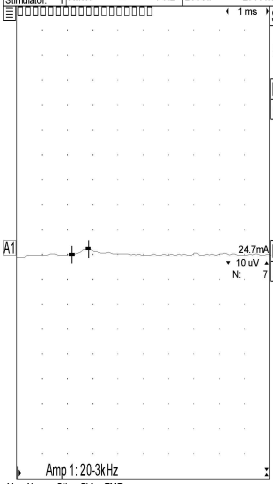

Recording Site: Lower leg

Stimulus Site	Lat1 ms	Lat2 ms	Amp uV
A1: Knee	2.2 U 2.8	2.8	2.5 L7.00

Segment	Dist mm	CV m/s
Lower leg-Knee	120	56 L54.0

New Nerve Other Side SNC

20.1
