# Supplementary material for: Case Report: Guillain−Barré syndrome temporally associated with levofloxacin exposure and improvement following efgartigimod treatment
Source: Front Immunol. 2026 Jan 7;16:1729694. doi: 10.3389/fimmu.2025.1729694 (PMC12819696; doi:10.3389/fimmu.2025.1729694)
Supplement: Supplementary file 2 [file DataSheet2.pdf]

The Second Affiliated Hospital of Guangxi Medical University
the Report on Electromyographic Evoked Potential (Day53)

Name：　　Sex：Female　　Age：23 y

ID：242200　　Department：EICU

Right Tibial MNC #35
Rec: AH, Stim: Ankle, Popliteal fossa — Record 16:55:44

Switch: STOP Stimulator: 1 | Rate: 1 Hz | Level: 27.4 mA | 0.2 ms | Single
Step: Hold | Average: Off | Sig. Enhancer: Off

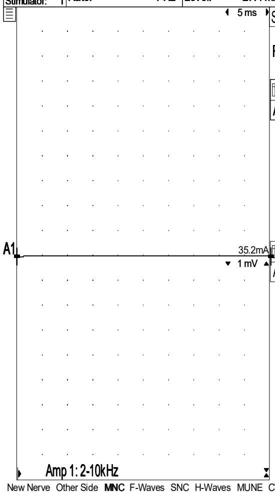

Recording Site: Abductor hallucis

Stimulus Site	Lat1 ms	Dur ms	Amp mV	Area mVms
A1: Ankle				

Segment	Dist mm	Diff ms	CV m/s
Abductor hallucis-Ankle			

Right Peroneal MNC #37
Rec: EDB, Stim: Ankle, Fibular head, PF — Record 16:55:55

Switch: STOP Stimulator: 1 | Rate: 1 Hz | Level: 27.4 mA | 0.2 ms | Single
Step: Hold | Average: Off | Sig. Enhancer: Off

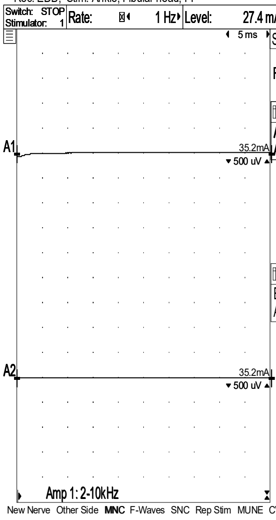

Recording Site: Extensor digitorum brevis

Stimulus Site	Lat1 ms	Dur ms	Amp mV	Area mVms
A1: Ankle				
A2: Fibula (head)				

Segment	Dist mm	Diff ms	CV m/s
Extensor digitorum brevis-Ankle			
Ankle-Fibula (head)			

Left Peroneal MNC #42
Rec: EDB, Stim: Ankle, Fibular head, PF — Record 16:56:05

Switch: STOP Stimulator: 1 | Rate: 1 Hz | Level: 27.4 mA | 0.1 ms | Single
Step: Hold | Average: Off | Sig. Enhancer: Off

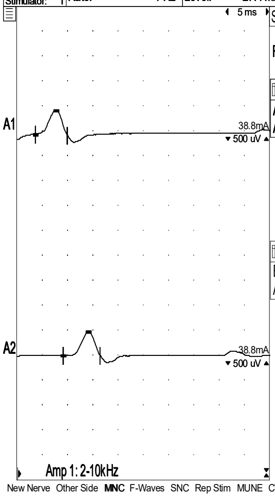

Recording Site: Extensor digitorum brevis

Stimulus Site	Lat1 ms	Dur ms	Amp mV	Area mVms
A1: Ankle	3.6	6.3	0.5	1.4
A2: Fibula (head)	9.1	7.3	0.5	1.3

Segment	Dist mm	Diff ms	CV m/s
Extensor digitorum brevis-Ankle		3.6	
Ankle-Fibula (head)	320	5.5	58

Left Tibial MNC #40
Rec: AH, Stim: Ankle, Popliteal fossa — Record 16:56:22

Switch: STOP Stimulator: 1 | Rate: 1 Hz | Level: 27.4 mA | 0.2 ms | Single
Step: Hold | Average: Off | Sig. Enhancer: Off

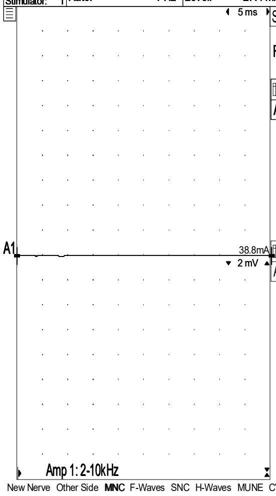

Recording Site: Abductor hallucis

Stimulus Site	Lat1 ms	Dur ms	Amp mV	Area mVms
A1: Ankle				

Segment	Dist mm	Diff ms	CV m/s
Abductor hallucis-Ankle			

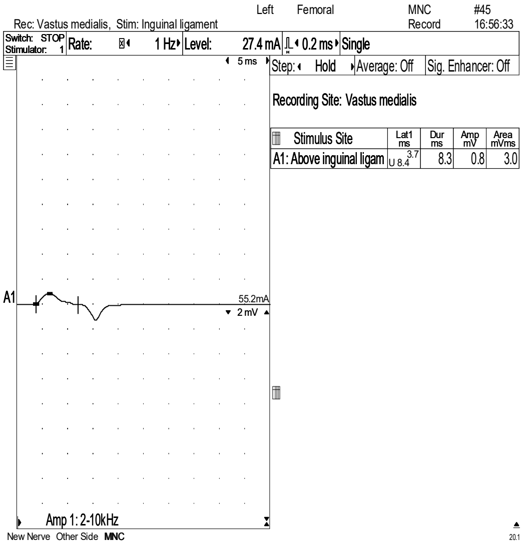
Left Femoral MNC #45
Rec: Vastus medialis, Stim: Inguinal ligament Record 16:56:33
Switch: STOP Stimulator: 1
Rate: 1 Hz
Level: 27.4 mA
0.2 ms
Single
5 ms
Step: Hold
Average: Off
Sig. Enhancer: Off
Recording Site: Vastus medialis
Stimulus Site
Lat1 ms
Dur ms
Amp mV
Area mVms
A1: Above inguinal ligam U 8.4 3.7 8.3 0.8 3.0
A1
55.2mA
2 mV
Amp 1: 2-10kHz
New Nerve Other Side MNC

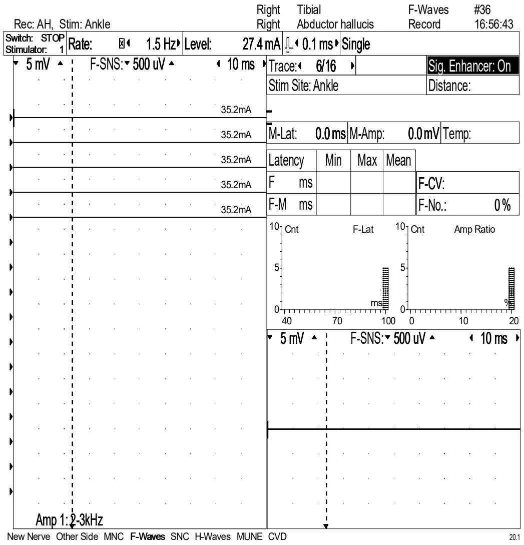
Right Tibial F-Waves #36
Rec: AH, Stim: Ankle Right Abductor hallucis Record 16:56:43
Switch: STOP Stimulator: 1
Rate: 1.5 Hz
Level: 27.4 mA
0.1 ms
Single
5 mV
F-SNS: 500 uV
10 ms
Trace: 6/16
Sig. Enhancer: On
Stim Site: Ankle
Distance:
35.2mA
M-Lat: 0.0 ms
M-Amp: 0.0 mV
Temp:
Latency
Min
Max
Mean
F ms
F-M ms
F-CV:
F-No.: 0 %
Cnt
F-Lat
Amp Ratio
Amp 1: 2-3kHz
New Nerve Other Side MNC F-Waves SNC H-Waves MUNE CVD

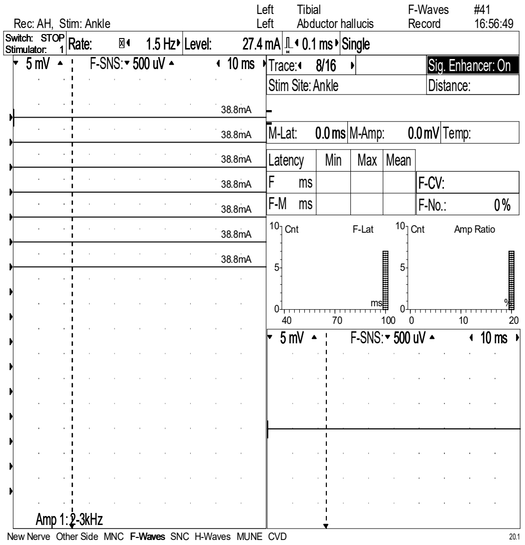
Left Tibial F-Waves #41
Rec: AH, Stim: Ankle Left Abductor hallucis Record 16:56:49
Switch: STOP Stimulator: 1
Rate: 1.5 Hz
Level: 27.4 mA
0.1 ms
Single
5 mV
F-SNS: 500 uV
10 ms
Trace: 8/16
Sig. Enhancer: On
Stim Site: Ankle
Distance:
38.8mA
M-Lat: 0.0 ms
M-Amp: 0.0 mV
Temp:
Latency
Min
Max
Mean
F ms
F-M ms
F-CV:
F-No.: 0 %
Cnt
F-Lat
Amp Ratio
Amp 1: 2-3kHz
New Nerve Other Side MNC F-Waves SNC H-Waves MUNE CVD

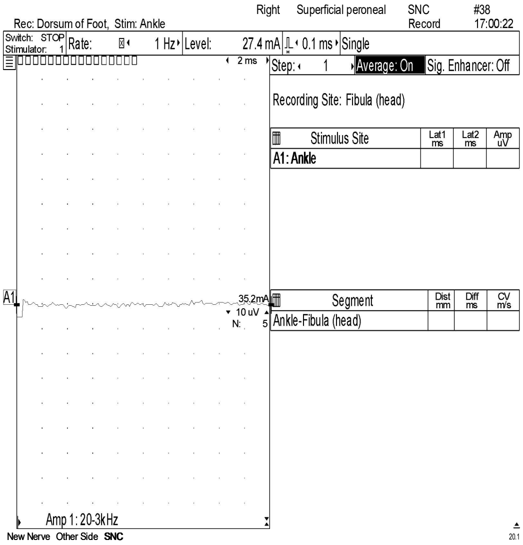
Right Superficial peroneal SNC #38
Rec: Dorsum of Foot, Stim: Ankle Record 17:00:22
Switch: STOP Stimulator: 1
Rate: 1 Hz
Level: 27.4 mA
0.1 ms
Single
2 ms
Step: 1
Average: On
Sig. Enhancer: Off
Recording Site: Fibula (head)
Stimulus Site
Lat1 ms
Lat2 ms
Amp uV
A1: Ankle
A1
35.2mA
10 uV
N: 5
Segment
Dist mm
Diff ms
CV m/s
Ankle-Fibula (head)
Amp 1: 20-3kHz
New Nerve Other Side SNC

Right Sural SNC #39
Rec: Lat. Mal., Stim: Calf (Antidromic) Record 17:00:26

Switch: STOP Stimulator: 1 | Rate: 1 Hz | Level: 27.4 mA | 0.1 ms | Single
Step: 1 | Average: On | Sig. Enhancer: On

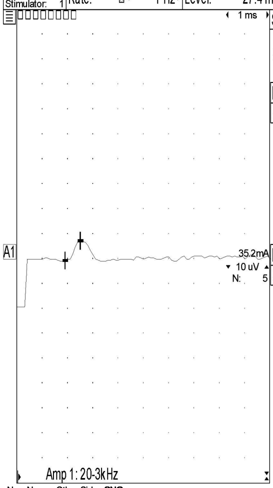

Recording Site: Soleus

Stimulus Site	Lat1 ms	Lat2 ms	Amp uV
A1: Lateral malleolus	1.9	2.5	8.4 L10.0

Segment	Dist mm	Diff ms	CV m/s
Soleus-Lateral malleolus	110	1.9	58 L41.0

New Nerve Other Side **SNC** 20.1

Left Superficial peroneal SNC #43
Rec: Dorsum of Foot, Stim: Ankle Record 17:00:31

Switch: STOP Stimulator: 1 | Rate: 1 Hz | Level: 27.4 mA | 0.1 ms | Single
Step: 1 | Average: On | Sig. Enhancer: On

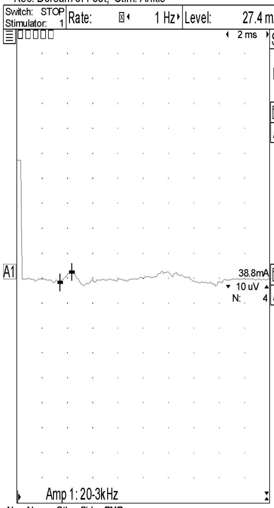

Recording Site: Fibula (head)

Stimulus Site	Lat1 ms	Lat2 ms	Amp uV
A1: Ankle	3.4	4.3	4.3 L10.0

Segment	Dist mm	Diff ms	CV m/s
Ankle-Fibula (head)	220	3.4	65 L40.0

New Nerve Other Side **SNC** 20.1

Left Sural SNC #44
Rec: Lat. Mal., Stim: Calf (Antidromic) Record 17:00:36

Switch: STOP Stimulator: 1 | Rate: 1 Hz | Level: 27.4 mA | 0.1 ms | Single
Step: 1 | Average: On | Sig. Enhancer: On

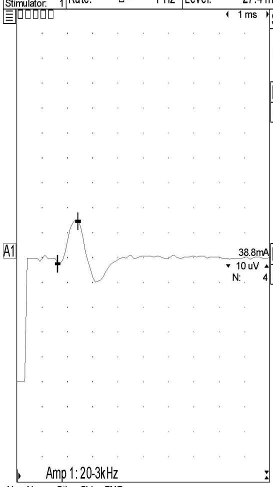

Recording Site: Soleus

Stimulus Site	Lat1 ms	Lat2 ms	Amp uV
A1: Lateral malleolus	1.6	2.4	17.7 L10.0

Segment	Dist mm	Diff ms	CV m/s
Soleus-Lateral malleolus	110	1.6	69 L41.0

New Nerve Other Side **SNC** 20.1

Right Saphenous SNC #46
Rec: Border of Tibia, Stim: Medial aspect knee Record 17:00:43

Switch: STOP Stimulator: 1 | Rate: 1 Hz | Level: 27.4 mA | 0.1 ms | Single
Step: 1 | Average: On | Sig. Enhancer: On

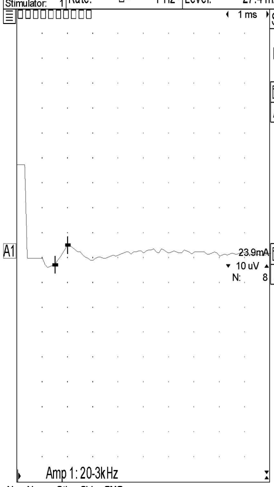

Recording Site: Lower leg

Stimulus Site	Lat1 ms	Lat2 ms	Amp uV
A1: Knee	1.5 U 2.8	2.0	8.3 L7.00

Segment	Dist mm	CV m/s
Lower leg-Knee	70	47 L54.0

New Nerve Other Side **SNC** 20.1
