# Supplementary material for: Case Report: Guillain−Barré syndrome temporally associated with levofloxacin exposure and improvement following efgartigimod treatment
Source: Front Immunol. 2026 Jan 7;16:1729694. doi: 10.3389/fimmu.2025.1729694 (PMC12819696; doi:10.3389/fimmu.2025.1729694)
Supplement: Supplementary file 3 [file Table1.docx]

| Supplementary Table 1 The scoring of Naranjo Adverse Drug Reaction (ADR) Probability Scale* | | | |
| --- | --- | --- | --- |
| Item No. | Questions | Response & Scoring | Scoring |
| 1 | Are there previous conclusive reports of this reaction? | Yes = +1, No = 0, Do not know = 0 | 1 |
| 2 | Did the adverse event appear after the suspected drug was administered? | Yes = +2, No = -1, Do not know = 0 | 2 |
| 3 | Did the adverse reaction improve when the drug was discontinued or a specific antagonist was administered? | Yes = +1, No = 0, Do not know = 0 | 1 |
| 4 | Did the adverse reaction reappear when the drug was readministered? | Yes = +2, No = -1, Do not know = 0 | 0 |
| 5 | Are there alternative causes (other than the drug) that could on their own have caused the reaction? | Yes = -1, No = +2, Do not know = 0 | -1 |
| 6 | Did the reaction reappear when a placebo was given? | Yes = -1, No = +1, Do not know = 0 | 1 |
| 7 | Was the drug detected in blood or other body fluids in toxic concentrations? | Yes = +1, No = 0, Do not know = 0 | 0 |
| 8 | Was the reaction more severe when the dose was increased, or less severe when the dose was decreased? | Yes = +1, No = 0, Do not know = 0 | 0 |
| 9 | Did the patient have a similar reaction to the same or similar drugs in the past? | Yes = +1, No = 0, Do not know = 0 | 0 |
| 10 | Was the adverse reaction confirmed by any objective evidence? | Yes = +1, No = 0, Do not know = 0 | 1 |
| *Total Score Range Causality Classification: ≥ 9, Definite; 5 - 8, Probable; 1 - 4, Possible; ≤ 0, Doubtful. | | | |
